# Software defect prediction using hybrid model (CBIL) of convolutional neural network (CNN) and bidirectional long short-term memory (Bi-LSTM)

**DOI:** 10.7717/peerj-cs.739

**Published:** 2021-11-16

**Authors:** Ahmed Bahaa Farid, Enas Mohamed Fathy, Ahmed Sharaf Eldin, Laila A. Abd-Elmegid

**Affiliations:** 1 Department of Information Systems, Faculty of Computers and Artificial Intelligence, Helwan University, Helwan, Egypt; 2 Department of Information Systems, Faculty of Computers and Artificial Intelligence, Beni-Suef University, Beni-Suef, Egypt; 3 Department of Information Systems, Faculty of Information Technology and Computer Science, Sinai University, Sinai, Egypt

**Keywords:** Defect, Software defect prediction, Abstract syntax tree, Machine learning, Deep learning, Convolutional neural network, Bidirectional long short-term memory

## Abstract

In recent years, the software industry has invested substantial effort to improve software quality in organizations. Applying proactive software defect prediction will help developers and white box testers to find the defects earlier, and this will reduce the time and effort. Traditional software defect prediction models concentrate on traditional features of source code including code complexity, lines of code, etc. However, these features fail to extract the semantics of source code. In this research, we propose a hybrid model that is called CBIL. CBIL can predict the defective areas of source code. It extracts Abstract Syntax Tree (AST) tokens as vectors from source code. Mapping and word embedding turn integer vectors into dense vectors. Then, Convolutional Neural Network (CNN) extracts the semantics of AST tokens. After that, Bidirectional Long Short-Term Memory (Bi-LSTM) keeps key features and ignores other features in order to enhance the accuracy of software defect prediction. The proposed model CBIL is evaluated on a sample of seven open-source Java projects of the PROMISE dataset. CBIL is evaluated by applying the following evaluation metrics: *F*-measure and area under the curve (AUC). The results display that CBIL model improves the average of *F*-measure by 25% compared to CNN, as CNN accomplishes the top performance among the selected baseline models. In average of AUC, CBIL model improves AUC by 18% compared to Recurrent Neural Network (RNN), as RNN accomplishes the top performance among the selected baseline models used in the experiments.

## Introduction

As software systems are evolving rapidly, software testing represents the most important phase in the development life cycle. Proactive software testing plays a master role in finding software defects early from the beginning of building the software system (Garousi et al., 2020). Software organizations employ code review and unit tests to improve code reliability and quality. However, those activities are time and resources consuming (Iki et al., 2021). Therefore, software defect prediction models are utilized to reveal the defective code automatically. Software defect prediction models employ the historical data of software then feed them to the model to predict code areas that contain defects (Wang, Zhuang & Zhang, 2021).

**Figure 1:**
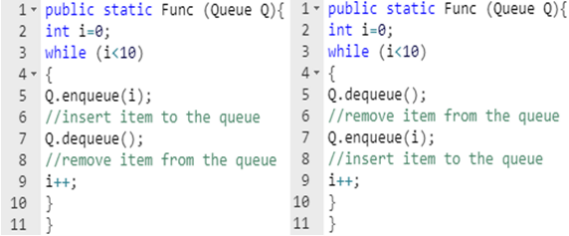
Two examples of clean and defective code.

Previous research studies (Hammouri et al., 2018; Wu et al., 2018; Wójcicki & Dabrowski, 2018; Agrawal & Menzies, 2018) that apply software defect prediction models categorize them as two approaches: the first one is concentrating on the traditional features of source code including lines of code, average method complexity, etc. However, traditional features fail to extract semantic information of programs because two programs have different semantics that may have the same values of the traditional features. For instance, Fig. 1 represents two Java source code: File1 and File2. The two files contain a while loop, and enqueue(i) function to insert items into the queue, and dequeue() function to delete items from the queue. To use the traditional features to represent the two examples, the two cases have identical traditional features because both have the same number of lines of code and the same functions, etc. However, File2 has a defect in the case of calling the dequeue function at the beginning of the code, and the queue is empty.

So, some recent research studies (Yang et al., 2015; Wang, Taiyue & Tan, 2016; Wang et al., 2017; Fan et al., 2019) propose the second approach that focuses on identifying how to extract semantic representation of programs.

Recently, deep learning has achieved a major progress to extract semantic features automatically and to improve software defect prediction accuracy and performance. Programs contain well-defined syntactic structure and semantic information hidden in two program representations (Li et al., 2019b). The first one is Abstract Syntax Tree (AST), and the second is Control Flow Graph (CFG). AST representation is chosen in the research studies of software defect prediction rather than CFG as it keeps the detailed information of source code.

We propose the model CBIL that combines both CNN and Bi-LSTM. The research is organized as follows: ‘Background’ introduces the background of software defect prediction and deep learning models. ‘Related Work’ reviews the related work about software defect prediction based on traditional features and defect prediction based on deep learning models. ‘The Proposed Approach’ describes the proposed model CBIL. The environment and experiments are shown in Environment and Experiments. Results are discussed in ‘Results’. The conclusion is discussed in ‘Conclusion and Future Work’.

## Background

### Software defect prediction

Software defects occur because human beings make mistakes. These mistakes will be converted to defect (fault, bug) in the code. If the code is executed, the software will fail to do the right behavior, causing failure to be appeared (Rahim et al., 2021). No software is bug-free. Most applications have many defects; they are classified as Critical, High, Medium, and Low. They can cause minor or major problems (Dhavakumar & Gopalan, 2021).

**Figure 2:**
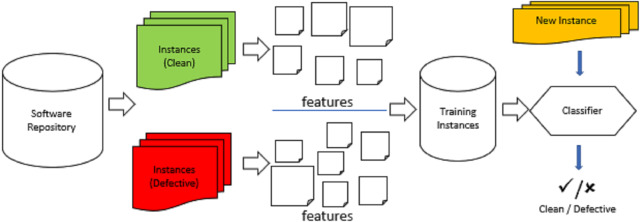
Software defect prediction process.

Software defect prediction is applied for predicting the areas that have defects in future releases (Tong, Liu & Wang, 2017). Figure 2 presents the steps of software defect prediction which are used in the research studies (Wang & Qiao, 2019; Zhou et al., 2019; Liang et al., 2019). There are four steps: the first one is to choose the suitable repositories. Then label the data as defective if it contains defects, otherwise, it is labeled as clean for each file. The second step is to extract and collect the key features of each file. The third step is to build and train the model by the labeled data and extracted features. At last, the classifier is used to predict if the new instance is defective or clean. There are two datasets in the model: training set is used to build and train the model, and test set is used to assess the trained model.

There are two kinds of software defect prediction models. The first one is Within-Project Defect Prediction (WPDP), and the second is Cross-Project Defect Prediction (CPDP) (Ni et al., 2019). In WPDP, the data are selected from historical versions in the same project. Both training and test sets are chosen from the same project. In CPDP, there are two different projects where the model is trained by the training set from one project, and the test set is from another project.

In this research, the performance of CBIL is evaluated on both WPDP and CPDP.

### Deep Learning (DL) (Ertel, 2017)

• Artificial Intelligence enables the machines to act as human behaviors.
• Machine Learning is a subset of Artificial Intelligence that enables the machines to learn from their experience.
• Deep Learning is a subset of Machine Learning that enables neural networks to act like the human brain, Deep means it uses a huge amount of data to train the model. Deep Learning consists of main models: Deep Belief Network (DBN), Convolutional Neural Network (CNN), and Recurrent Neural Network (RNN).

#### CNN

CNN (Li et al., 2019a) is a particular kind of neural network that is used in the areas such as Natural Language Processing (NLP), speech recognition, and text classification. CNN (Meilong et al., 2020) is categorized into one-dimensional (1D) CNN for NLP and two-dimensional (2D) CNN for image recognition.

**Figure 3:**
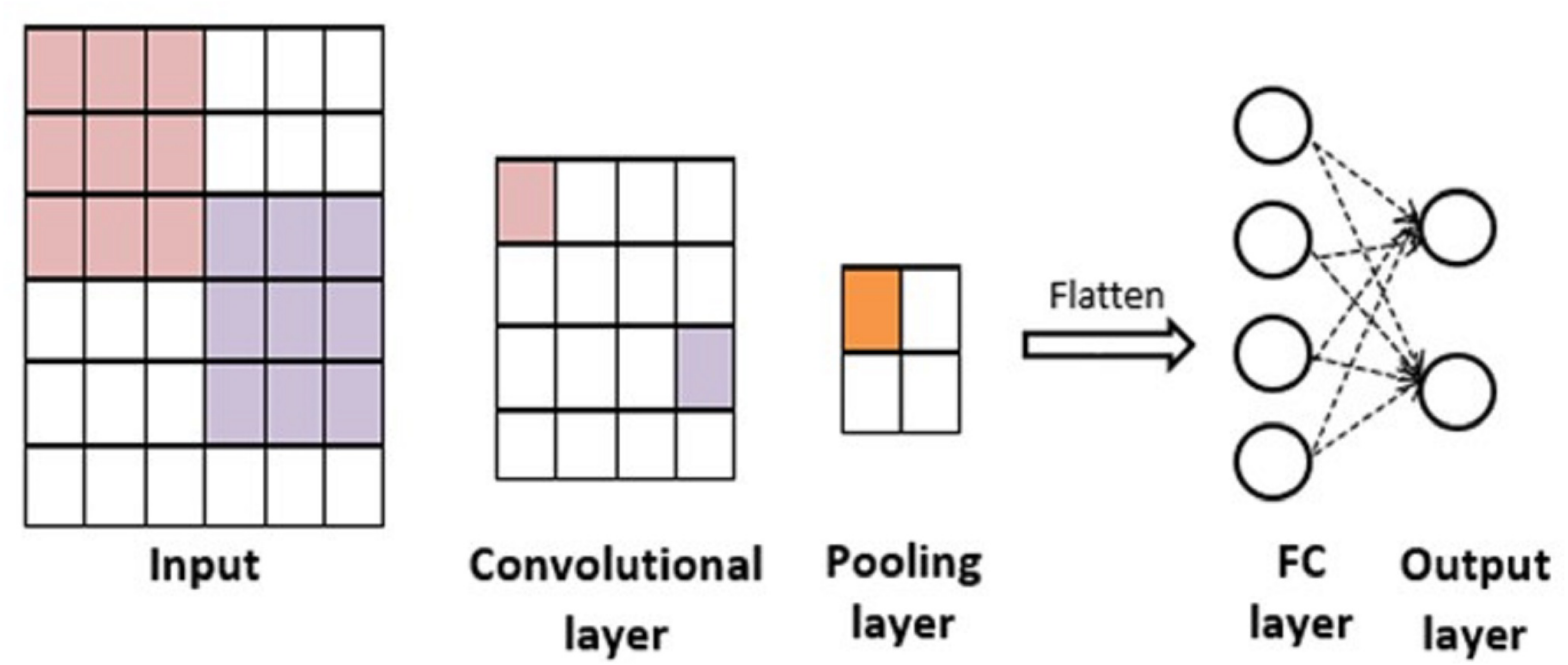
CNN architecture.

Simple CNN architecture (Dahou et al., 2019) is shown in Fig. 3. The number of convolutional filters is defined in the convolutional layer. It is applied to input data for producing feature maps. Then, CNN uses the pooling layer to minimize the dimensionality of the output. Max pooling is mostly used as it accomplishes better effectiveness compared to both Min pooling and Average pooling techniques (Goncalves dos Santos, 2020). Then, flatten layer transforms a 2D matrix into a vector that can be used as input to a fully connected (FC) layer. At last, FC layer and sigmoid represent the output layer, it generates the results of the classification based on previous layers.

CNN has two key features (Sheng, Lu & Lin, 2020): sparse connectivity and shared weights. These features minimize the capacity of the model and catch global manners rather than local ones.

#### RNN

RNN (Sherstinsky, 2018), it is a sequential model, a generalization of feedforward neural network with internal memory to save the states of every input in the network. In a feedforward neural network, the information goes through only one direction, while RNN can store information over time. The loops in RNN called recurrent because the information is passed internally from a one-time step to the next time step. RNN (Deng, Lu & Qiu, 2020) has been achieved a good progress in many fields, including sequence recognition, sequence reproduction, and temporal association. RNN (Zhang, Chen & Huang, 2018) takes input vector as X_t_, Y_t_ represents the output then the calculations are executed to update an internal state h_t_. The following equation is executed:

(1) \begin{eqnarray*}{h}_{t}=fw({h}_{t-1},{X}_{t})\end{eqnarray*}

Where *f*w is the activation function with a set of w weights, h_*t*−1_ represents the old state, and X_t_ is the input vector at time step t. In RNN, the same procedures are used at every step.

To update the internal state by applying activation function (tanh), the following equation is executed:

(2) \begin{eqnarray*}{h}_{t}=\tanh \nolimits ({w}_{hh}{h}_{t-1}{+w}_{xh}{X}_{t})\end{eqnarray*}

Where w_hh and_ w_xh_ weights of internal state and input.

The output will be calculated as the following equation:

(3) \begin{eqnarray*}{Y}_{t}={w}_{hy}{h}_{t}\end{eqnarray*}

Where w_hy_ the weight of the output layer.

**Figure 4:**
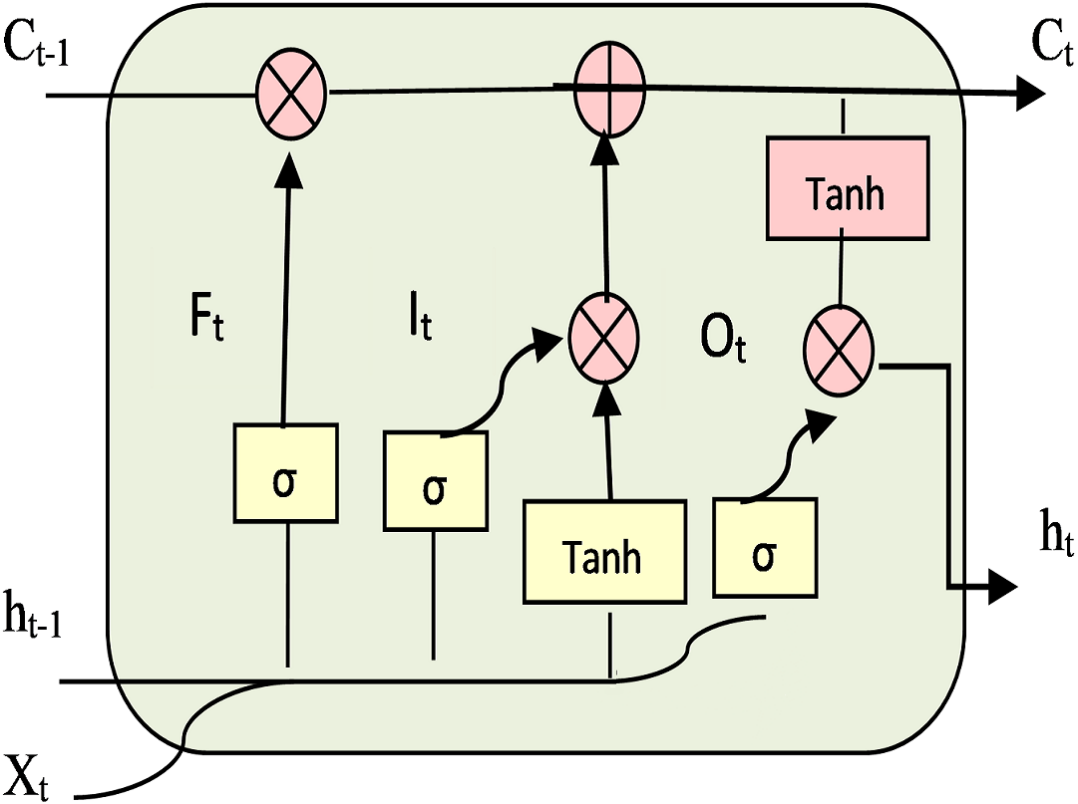
Framework of LSTM units.

A common problem in RNN is the “vanishing gradient” problem (Gao et al., 2020), where the gradients become extremely small, and it might be impossible to make any optimization. Long Short-Term Memory (LSTM) (Ralf & Rothstein Morris, 2019) is a type of RNN that is used to solve the vanishing gradient. LSTM contains a “memory cell” that can track and maintain information of long-term dependencies in memory for very long periods of time. LSTM units (Wu et al., 2020) consist of three gates: input gate, forget gate, and output gate. Figure 4 shows the framework of standard LSTM units, where *σ* is a logistic sigmoid function, Tanh is the activation function, X_t_ represents new input, h_*t*−1_ represents output from previous timestep, h_t_ is the output, old cell state C_*t*−1_, new cell state C_t_. Also, *F*_t_, I_t_, O_t_ symbolize forget gate, input gate, and output gate respectively.

The gates enable the information to be added or removed at a cell state. The data will be passed through a sigmoid function. The sigmoid function is forcing the input to be between 0 and 1. The information will be passed if the value equals 1 only. Forget gate performs computations to store relevant history of new information to cell state and discards the irrelevant history from the cell state. Then input gate controls if the cell state is updated or not. Also, the output gate handles the value in the cell state. Then the activation of LSTM unit will be calculated. The following equations are used to compute h_t:_

(4) \begin{eqnarray*}{F}_{t}& =\sigma ({W}_{f}{X}_{t}+{U}_{f}{h}_{t-1}+{b}_{f})\end{eqnarray*}

(5) \begin{eqnarray*}{I}_{t}& =\sigma ({W}_{i}{X}_{t}+{U}_{i}{h}_{t-1}+{b}_{i})\end{eqnarray*}

(6) \begin{eqnarray*}{O}_{t}& =\sigma ({W}_{o}{X}_{t}+{U}_{o}{h}_{t-1}+{b}_{o})\end{eqnarray*}

(7) \begin{eqnarray*}{C}_{t}^{\wedge }& =\tanh \nolimits ({W}_{c}{X}_{t}+{U}_{c}{h}_{t-1}+{b}_{c})\end{eqnarray*}

(8) \begin{eqnarray*}{C}_{t}& =ft\Theta {C}_{t-1}+{I}_{t}\Theta {C}_{\mathrm{t}}^{\wedge }\end{eqnarray*}

(9) \begin{eqnarray*}{h}_{t}& ={O}_{t}\Theta \tanh \nolimits ({c}_{t})\end{eqnarray*}

Where *b* is the bias, *W* and *U* are the weights of the three gates. *C*^∧^_t_ is the candidate value. Θ is the multiplication operation; it determines if the information will pass through the gates or not.

Bi-directional LSTM (Bi-LSTM), It combines two independent LSTM together. It is useful for sentiment classification. It enables the information to be passed through forward and backward directions.

## Related Work

Software defect prediction is a crucial point in software engineering. We have published a Systematic Literature Review in software defect prediction using deep learning (Bahaa et al., 2021). The goal of this SLR is to identify the research studies that apply the semantic features of the source code. This SLR helps us to know the gaps in software defect prediction studies. And, to propose a novel deep learning model which can improve the prediction of software defects. We analyze Forty primary studies based on the selected quality criteria. The results show that most of the studies apply WPDP by 52.5%. However, some deep learning models achieve good results with WPDP and very bad results with CPDP. Also, few studies propose the hybrid deep learning models. Regarding to the evaluation metrics, most of the studies apply one metric. However, more and more evaluation metrics should be selected to evaluate the performance of the proposed models.

A considerable number of studies of this field concentrate on traditional features that are included in static code metrics. Mousavi, Eftekhari & Rahdari (2018) introduced a model that merges over bagging and ensemble learning approaches to fix the problem of class imbalance in training and test data sets. Agrawal & Menzies (2018) defined how to check the performance of classifiers under multiple criteria. And they proposed a data preprocessing technique to repair the weakness in the training data. They concluded that data preprocessing is more required than the type of classifiers. Wójcicki & Dabrowski (2018) found most studies ensure that the models could be applied for C, C++, and Java projects only. So, they tried to emphasize that the previous models also could be used to python projects. Moreover, Hammouri et al. (2018) proposed a combined model that includes multiple supervised machine learning classifiers. These classifiers are applied to predict the defects based on the stored data.

The problem occurred in the insufficient information in the WPDP. So, some research studies concentrated on CPDP. Wu et al. (2018) focused on the dictionary learning technique. They covered two categories of defect data: limited labeled data and plenty of unlimited data to be used in the kernel space. Qiu, Lu & Jiang (2018) introduced a model to analyze various components of each source project to build an optimized ensemble classifier for a target project. Traditional features of source code were used as input to software defect prediction models in all the previous studies. However, these features cannot extract the semantic information of programs.

Recently, deep learning models have been used to enhance the prediction of software defects. Yang et al. (2015) introduced a deep learning model by using the DBN to produce new features from current features. After that, the new features were used to foresee the defective changes. They used fourteen basic change features such as code added, code deleted, line of code before the change, line of code after the change, the modified files, and the modified directories. Wang, Taiyue & Tan (2016) introduced the DBN model to capture the semantics from AST tokens in each program. Then these semantics were entered into the classifier to foresee the defective code for both WPDP and CPDP at the file level. Wang et al. (2017) worked on their research (Wang, Taiyue & Tan, 2016) and proposed an updated DBN model to pick up the semantic representation of the software from both of source code and code changes. Source code was used for file-level, and code changes for change-level.

As noticed from deep learning research studies in most fields, CNN is better than DBN since it can capture context and semantics more effectively. Li et al. (2017) presented a hybrid model that is called “Defect Prediction via Convolutional Neural Network” (DP-CNN), by merging extracted semantic features from CNN and traditional features like lines of code, weighted methods per class, etc. Khanh et al. (2018) proposed a deep learning model, which was built on LSTM, it takes a raw AST of a source file then predicts if the file is defective or clean. Lin et al. (2018) proposed a Bi-LSTM model to extract code semantics from serialized AST. Then applied it with continuous bag of words and fed the serialized AST into Bi-LSTM to discover the most probable vulnerable functions. Liang et al. (2019) proposed a frame that merges LSTM model and unsupervised word embedding, by using AST tokens. Then mapping every token into a real-valued vector. Furthermore, using the vectors and their labels to build the LSTM to pick up the semantic information of the software. Fan et al. (2019) introduced a defect prediction framework that was built on an Attention-based Recurrent Neural Network. They employed ASTs to be used as vectors, then applied dictionary mapping and word embedding to allow the framework to learn syntactic and semantic features automatically. At last, Attention Mechanism was selected to generate the key features for enhancing the defect prediction.

Unlike these research studies, CBIL combines both of CNN and Bi-LSTM. The strength of CBIL as follows:

1. CNN extracts as many semantic features as possible from AST tokens.

2. Bi-LSTM keeps the chronological order between AST tokens and detects information of long-term dependencies, so it can keep key features and ignore unnecessary features.

## The Proposed Approach

**Figure 5:**
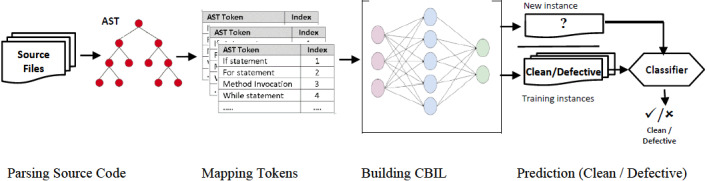
Overview of CBIL model.

In this section, the CBIL model is presented. The overall framework is shown in Fig. 5. It extracts the AST tokens from the Abstract Syntax Tree for all java files in the training and test sets. Then, the vector of text tokens is converted to an integer vector by building a mapping dictionary among tokens and integers. Then, word embedding is employed to turn each integer vector to a dense vector. At last, the generated dense vector is used as input to build and train CBIL model. Then its performance is evaluated on the PROMISE dataset.

CBIL consists of two phases:

1. Data Preprocessing.
2. Building CBIL Model.

### Data preprocessing

In this phase, we prepare the data before building the model to improve its accuracy and to resolve the class imbalance issue which may affect negatively on the results of the model.

#### Parsing source code into AST

There are two main steps to use the source code of the files in the model:

First, Abstract Syntax Tree (AST) is built for the selected Java files included in PROMISE dataset.

**Figure 6:**
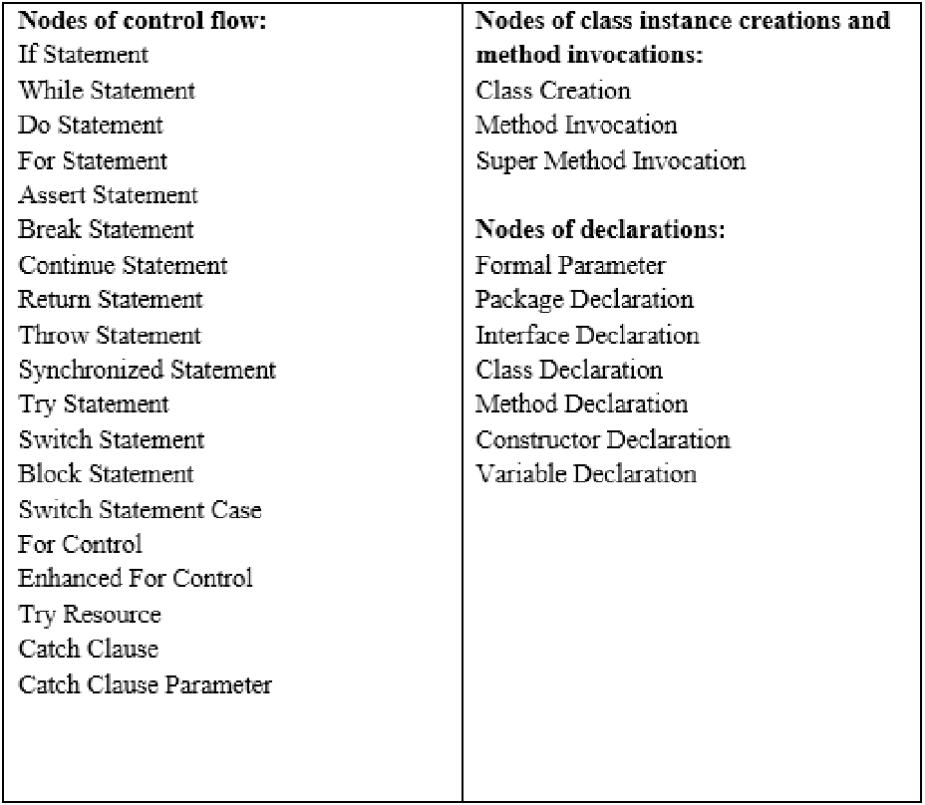
Selected AST nodes.

Second, the following kinds of AST nodes are selected to be generated as tokens: (1). nodes of control flow such as if/while/do statements which are recorded as the types of the nodes. (2). nodes of class instance creations and method invocations which are recorded as the names of classes or methods without Parenthesis (3). nodes of declarations such as method/class declarations which are recorded as their names. Other AST nodes are removed as it may affect the importance of the selected AST nodes. The selected AST nodes are shown in Fig. 6. We apply the Python package “javalang” to parse the source code into AST.

#### Mapping tokens

When the source code is parsed, text tokens are extracted for every file. But it is difficult to use them directly as input into DL model. So, it is needed to convert the text tokens to an integer vector. The mapping between the tokens and the integers is built with the range of one to the total number of tokens. So, a unique integer will represent each token. “Keras Text tokenization utility” class is applied to convert all text tokens to a sequence of integers. All integer vectors should have fixed length. Identified length will be selected. So, if the vector length is smaller than the identified length, it is completed by 0. And, If the vector length is bigger than the identified length, the extra length will be removed.

#### Class imbalance

The data are imbalanced most of the time, as instances of defective files are less than the number of instances of clean files. Therefore, the prediction results will be classified as clean as the instances of clean files represents the majority class. To resolve the class imbalance issue (Eivazpour & Keyvanpour, 2021), there are two common techniques to be used: oversampling and undersampling. In oversampling, the instances are duplicated in the minority class. In undersampling, the instances will be deleted from the majority class. To ensure the completeness of data, oversampling technique is applied by using “imblearn RandomOverSampler” method. It will duplicate the instances of minority class randomly over time.

### Building CBIL model

**Figure 7:**
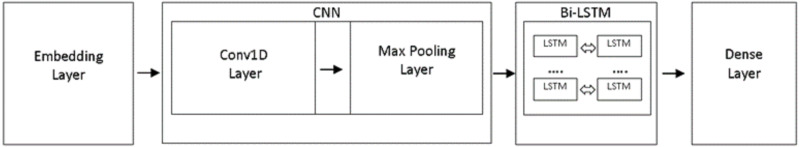
CBIL layers.

Figure 7 shows the layers of CBIL model. CBIL model combines the advantages of both CNN and Bi-LSTM. CNN extracts semantic features of AST tokens. Bi-LSTM can preserve the sequential order between the data. And it can deal with long sequences of tokens. The model consists of four layers: an embedding layer, CNN layers, Bi-LSTM layer, and dense layer.

#### Embedding layer

Integer vectors cannot carry the context information of AST tokens. Therefore, word embedding (Yildiz & Tezgider, 2021)technique is used to turn each integer vector to a dense vector. In traditional embeddings, large sparse vectors are used to represent each word within a vector to represent an input sequence. However, this representation is sparse because the input sequences are huge. For example, AST tokens will be preserved while the relationship between them will be ignored. Instead, in word embedding, words are represented using dense vectors with a fixed length where a vector represents the word’s projection into a continuous vector space. For example, a “For Statement” node is embedded to dense vector [0.25,0.1]. Word embedding has two main benefits. Firstly, the embedded vector has lower dimensions than the sparse vector. Secondly, AST nodes that share identical contexts are located near each other in the vector space.

#### CNN layers

It includes two layers: convolutional layer and max pooling layer. In convolutional layer, it is applied to automatically learn the features of software defects. It extracts the key semantics based on number of filters and filter length. We analyze the values of filter length and number of filters to choose the optimal values. The experiments have been conducted on project Jedit, Lucene, Poi and Xalan as a sample of the selected projects. Filter length is set to 10, and number of filters to 20. The details of the optimal values are discussed in ‘Results’. Then, max pooling is applied to reduce the dimensionality of the output. It is used to gather the extracted information. The results of max pooling are entered as input to Bi-LSTM layer to filer the useful information.

#### Bi-LSTM layer

It preserves the sequential order between the data. The implementation of Bi-LSTM helps to detect the information of long-term dependencies which can successfully capture necessary features. It runs two LSTMs to detect both of forward and backward information. We analyze the value of LSTM units to choose the optimal value. The experiments have been conducted on project Jedit, Lucene, Poi and Xalan as a sample of the selected projects. The number of LSTM units is set to 32. The detail of the optimal value is discussed in ‘Results’.

#### Dense layer

Finally, the Bi-LSTM layer is connected to the dense layer to obtain the results of the prediction. CBIL model is trained by using a training set based on their labels (i.e., defective, or clean). Then, Logistic Regression classifier is applied on the test set to generate the probability of the file being defective.

## Environment and Experiments

Several experiments are applied to study the effectiveness of the proposed model CBIL. Then the results are compared with other ML and DL models used in software defect prediction. The experiments are implemented on Python3.8, Keras2.3.1, TensorFlow2.1.0, scikit-learn0.22.1, numpy1.18.1, and imbalanced-learn0.3.2, with server running Ubuntu 18.04.4 LTS with processor Intel^®^ Core™ i7-8550U CPU @ 1.80 GHz and RAM of 16 GB. The following parameter settings are used for CBIL:

• Embedding dimension is set to 30 and the length of AST vector is set to 2000.
• In CNN layers, the value of filter length is 10 and the number of filters is 20. These values are selected based on the optimal parameter settings discussed in ‘Results’, with applying tanh activation function.
• In Bi-LSTM layer, the number of LSTM units is set to 32 as discussed in ‘Results’, with applying tanh activation function.
• RMSprop is the optimizer that is used in the experiments, and binary_crossentropy is used for loss function.
• Sigmoid activation function is used in the dense layer.
• All the above parameters are applied on a batch size of 32 and epoch of 40.

### Datasets

Seven open-source projects are chosen written in Java language extracted from PROMISE dataset. It is a public repository used for the prediction of software defects. These projects cover several applications such as XML parser, text search engine library, and data transport adapters. The details of each project are shown in Table 1, including project name, two releases of each project, their total files, and the defects rate for them. Each project has two releases, first release is used for training the model and second release for evaluating it. In PROMISE, the projects have traditional features for each Java file. All traditional features are shown in Table 2.

### Evaluation metrics

The performance of CBIL model is evaluated by *F*-measure and AUC (area under ROC (receiver operating characteristic) curve). These evaluation metrics (Jayanthi & Florence, 2019; Saharudin, Wei & Na, 2020) are vastly used to assess the previous research studies of software defect prediction models. *F*-measure is calculated as follows:

(10) \begin{eqnarray*}Precision& = \frac{TP}{ \left( TP+FP \right) } \end{eqnarray*}

(11) \begin{eqnarray*}Recall& = \frac{TP}{ \left( TP+FN \right) } \end{eqnarray*}

(12) \begin{eqnarray*}F-measure& = \frac{2\ast Precision\ast Recall}{Precision+Recall} \end{eqnarray*}

The beginning with confusion matrix; it is used to describe ML and DL models’ performance. It encapsulates the prediction results of the model. Also, it generates the results of the following classes: True Positive (TP), False Positive (FP), True Negative (TN), and False Negative (FN).

Precision and Recall are calculated by three classes of the confusion matrix (TP, FP, FN) where TP means number of predicted defective files that are already defective, FP means number of predicted defective files but there are clean files, and FN means number of predicted clean files that are defective. It is very complicated to compare two models with high Recall and low Precision or vice versa. Therefore, it is needed to use more powerful metric as *F*-measure. The range of *F*-measure is from 0 to 1 where the higher value means the better effectiveness of the model.

**Table 1:** Description of the PROMISE dataset.

Project	Releases	Total files	Defect’s rate (%)
Camel	1.4, 1.6	1837	18.13
Jedit	4.0, 4.1	618	24.92
Lucene	2.0, 2.2	442	53.17
Poi	2.5, 3.0	827	63.97
Synapse	1.1, 1.2	479	30.48
Xalan	2.5, 2.6	1688	47.27
Xerces	1.2, 1.3	893	15.68

**Table 2:** Traditional features used in the PROMISE dataset.

Traditional features	
Weighted methods per class	Lines of code
Depth of inheritance tree	Response for a class
Number of children	Data access metric
Coupling between object classes	Measure of aggregation
Measure of function abstraction	Afferent coupling
Lack of cohesion in methods	Efferent coupling
Cohesion among methods of a class	Inheritance coupling
Lack of cohesion in methods3	Average method complexity
Number of public methods	Coupling between methods
Maximum McCabe	Average McCabe

AUC (Lin & Lu, 2021) is focused on the area under the ROC curve. ROC curve displays the behavior of the model at all classification outsets. False Positive Rate (FPR) are represented in X axis of the ROC curve. while True Positive Rate (TPR) is represented in Y axis. Each classification outset generates a coordinate of (FPR, TPR), and all coordinates form a Roc curve. Range of AUC from 0 to 1 where the higher value means the better effectiveness of the model. AUC is calculated based on the following equations:

(13) \begin{eqnarray*}FPR& = \frac{FP}{ \left( FP+TN \right) } \end{eqnarray*}

(14) \begin{eqnarray*}TPR& = \frac{TP}{(TP+FN)} & \end{eqnarray*}

### Research questions

We deploy the experiments to evaluate the effectiveness of CBIL model. The following research questions (RQs) should be answered:

**RQ1**: Do deep learning models enhance the performance of WPDP rather than traditional machine learning models which based on traditional features?

**RQ2**: Does CBIL achieve better performance for WPDP more than other deep learning models?

**RQ3**: What is the optimal performance of CBIL for WPDP under different parameter settings?

**RQ4**: What is the behavior of CBIL for both WPDP and CPDP?

### Baseline models

The proposed model CBIL is compared with the following baseline models:

1. Traditional: the most common of traditional machine learning models is Random Forest (RF Kumar et al., 2021).
2. DBN (Zhang & Liu, 2020): this is the first deep learning model which applied in software defect prediction. It automatically extracts semantic features from source code files.
3. CNN: this is the common deep learning model which based on convolutions to reduce the dimensionality of the output.
4. RNN: it is the most vital deep learning model used in NLP. It focuses on capturing the semantics to enhance the prediction of software defects.

The following parameter settings are used and the results for baseline models as in Fan et al. (2019) and Li et al. (2017). DBN has 10 hidden layers, each layer has 100 nodes, and the vector length is 2000. In CNN, 10 filters are chosen, and each filter has length of 5. In RNN, the parameters of embedding dimensions are set to 30, first hidden layer has 16 nodes with second layer has 24 nodes, and the vector length is 2000.

## Results

The results of CBIL model are presented in this section. Also, the research questions are answered. *F*-measure and AUC are shown in Tables 3 and 4. The tables summarize the comparison between CBIL and other baseline models. The best values of *F*-measure and AUC are highlighted in the two tables.

To answer RQ1, four deep learning models (DBN, CNN, RNN, CBIL) are compared with the selected machine learning model (RF). The experiments have been conducted on PROMISE dataset listed in Table 1. For each project, first release is used for training the model and second release for evaluating it. In Table 3, *F*-measure values are shown for each project generated by implementing CBIL and other baseline models. Project Poi is taken as a sample of all projects, *F*-measure values of RF, DBN, CNN, RNN, and CBIL are 0.669, 0.780, 0.778, 0.722 and 0.852 respectively. As shown in Table 3, the average of *F*-measure in deep learning models is better than the average of *F*-measure in traditional model. Table 4 shows the AUC values of each project. In most AUC values, deep learning models of DBN, CNN, RNN, and CBIL have high values of AUC rather than the traditional model. And also, the average of AUC in deep learning models is better than the average of AUC in the traditional model. In conclusion, deep learning models accomplish top performance than traditional machine learning models for software defect prediction.

**Table 3:** *F*-measure of CBIL and baseline models.

Project	RF	DBN	CNN	RNN	CBIL
Camel	0.396	0.335	0.505	0.515	**0.935**
Jedit	0.550	0.480	0.631	0.595	**0.850**
Lucene	0.604	0.758	**0.761**	0.672	0.744
Poi	0.669	0.780	0.778	0.722	**0.852**
Synapse	0.414	0.503	0.512	0.487	**0.889**
Xalan	0.638	0.681	0.676	0.606	**0.716**
Xerces	0.185	0.261	0.311	0.262	**0.951**
Average	0.494	0.543	0.596	0.551	0.848

**Table 4:** AUC of CBIL and baseline models.

Project	RF	DBN	CNN	RNN	CBIL
Camel	0.677	0.654	0.732	0.766	**0.963**
Jedit	0.797	0.794	0.841	0.842	**0.911**
Lucene	0.641	0.682	0.688	0.693	**0.833**
Poi	0.636	0.668	0.745	0.764	**0.951**
Synapse	0.682	0.657	0.632	0.648	**0.950**
Xalan	0.674	0.676	0.674	0.654	**0.769**
Xerces	0.576	0.560	0.671	0.730	**0.981**
Average	0.669	0.670	0.712	0.728	0.908

To answer RQ2, CBIL model is compared with three deep learning models (DBN, CNN, RNN). The experiments have been conducted on PROMISE dataset listed in Table 1. As before, first release is used for training the model and second release for evaluating it. In Table 3, *F*-measure values are shown for each project generated by implementing CBIL and other baseline models. CBIL accomplishes the best performance in six projects. The only project with lower value is Lucene, where CBIL achieves a lower *F*-measure than both of DBN and CNN. In average of *F*-measure, the best performance of deep learning models is CBIL, CNN, RNN then DBN respectively. Table 4 shows the AUC values of each project. The best value of AUC is achieved by CBIL in all the seven projects. In average of AUC, the order of deep learning models from the highest to the lowest is CBIL, RNN, CNN and DBN respectively. In conclusion, CBIL model accomplishes higher performance among all deep learning models.

**Figure 8:**
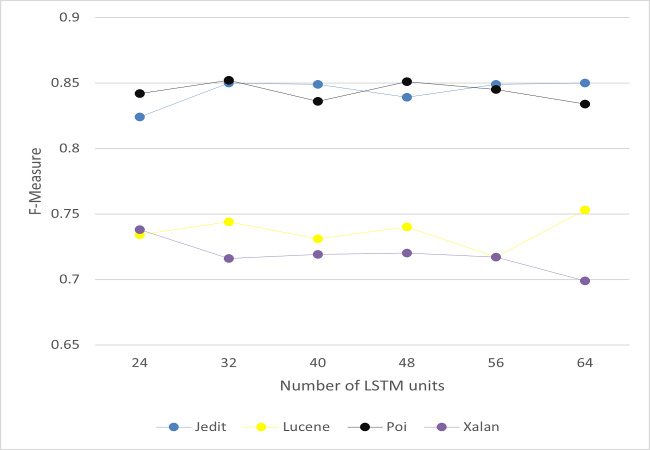
*F*-measure of CBIL under LSTM units.

**Figure 9:**
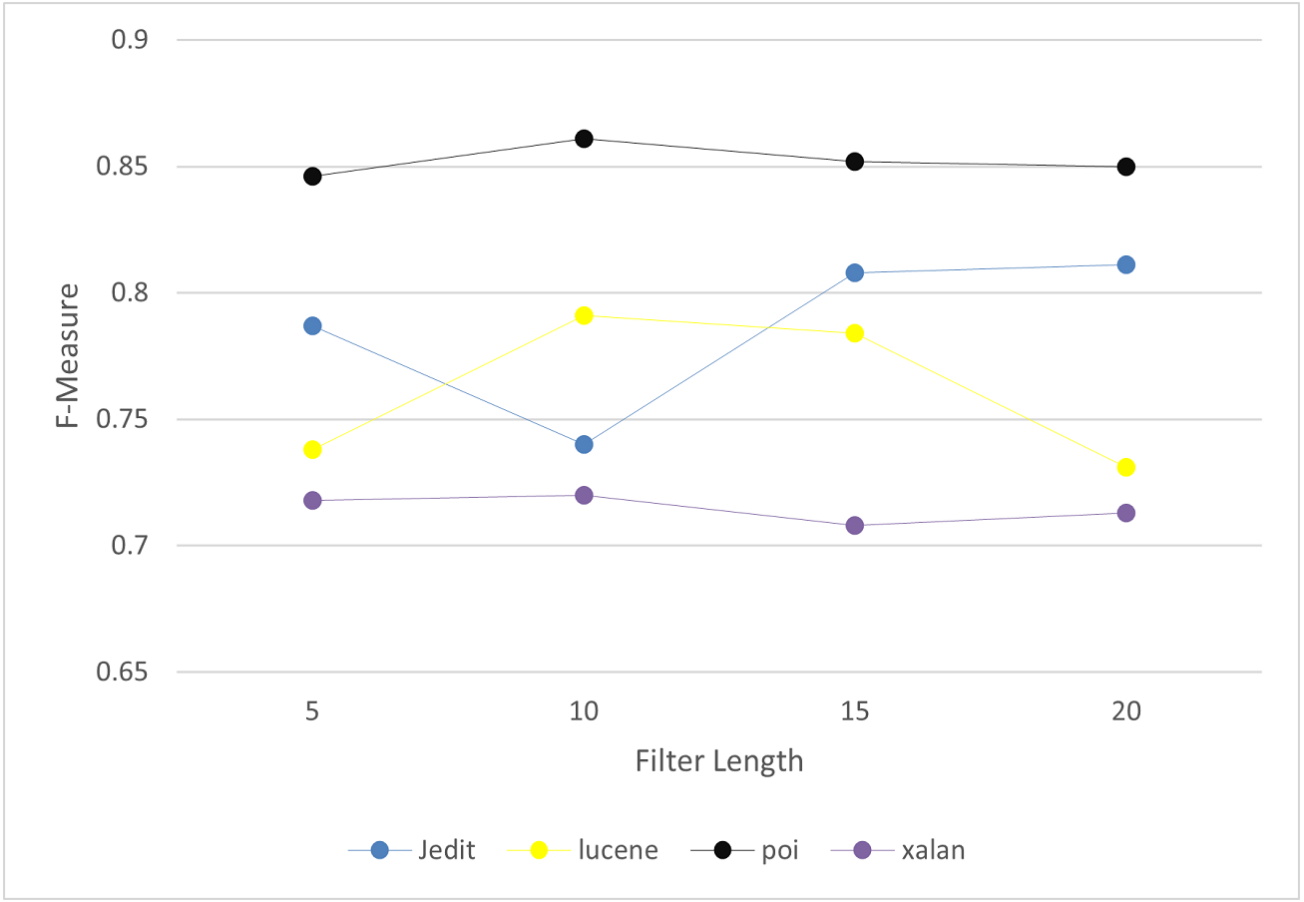
*F*-measure of CBIL under filter length.

**Figure 10:**
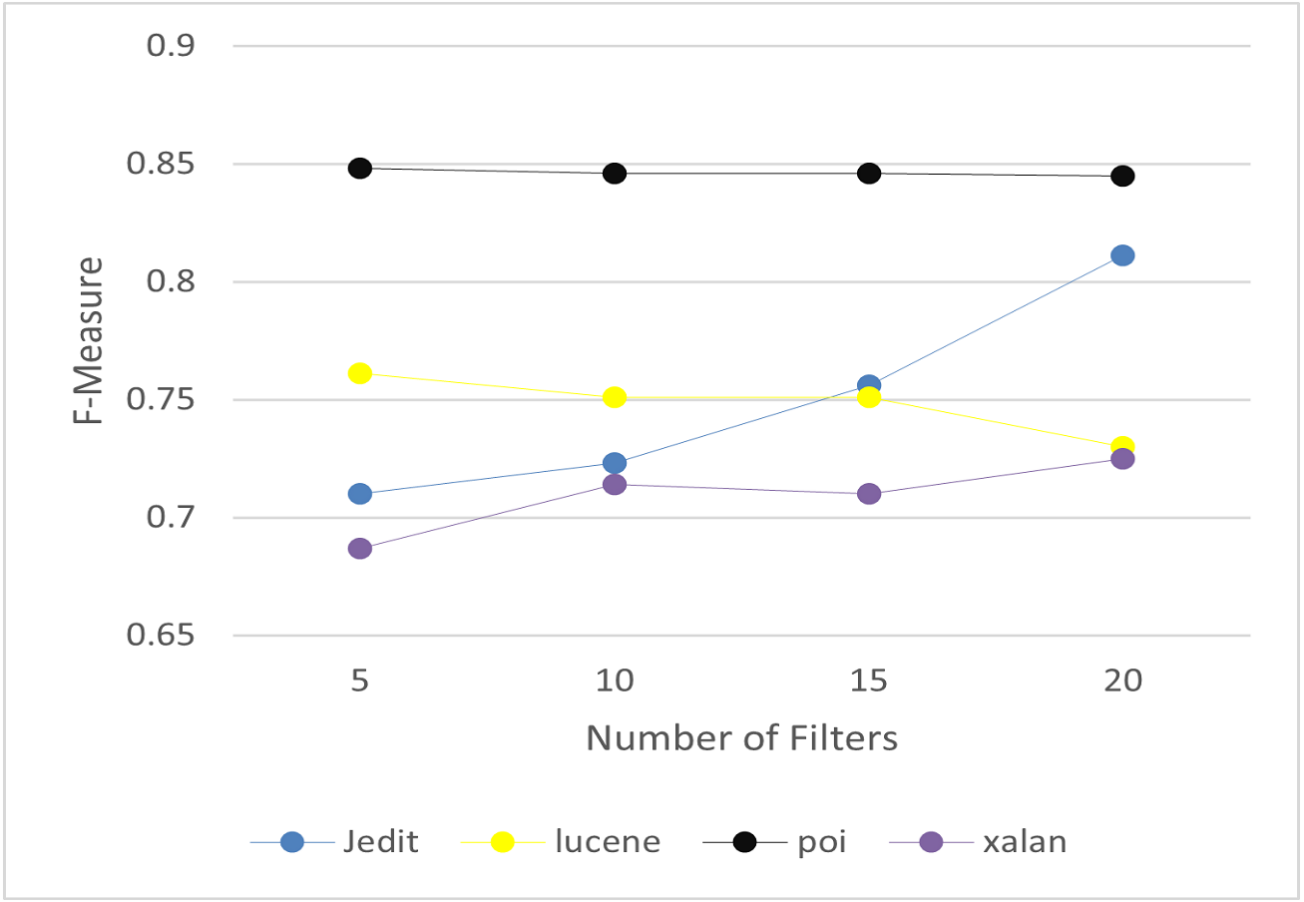
*F*-measure of CBIL under filters.

To answer RQ3, we set the key parameters in CBIL to obtain the best achievement and effectiveness. The experiments have been conducted on project Jedit, Lucene, Poi and Xalan as a sample of the seven projects. There are three key parameters in CBIL: the number of LSTM units, the filter length, and the number of filters. The average of *F*-measure is calculated for each project under different values of the three parameters to choose the values that achieve the best performance for software defect prediction. Figures 8–10 show the *F*-measure of CBIL under different number of LSTM units, filter length, and number of filters respectively. As shown in the figures, the optimal number of LSTM units is 32, filter length is 10, and number of filters is 20. So, these optimal values are used for CBIL.

**Figure 11:**
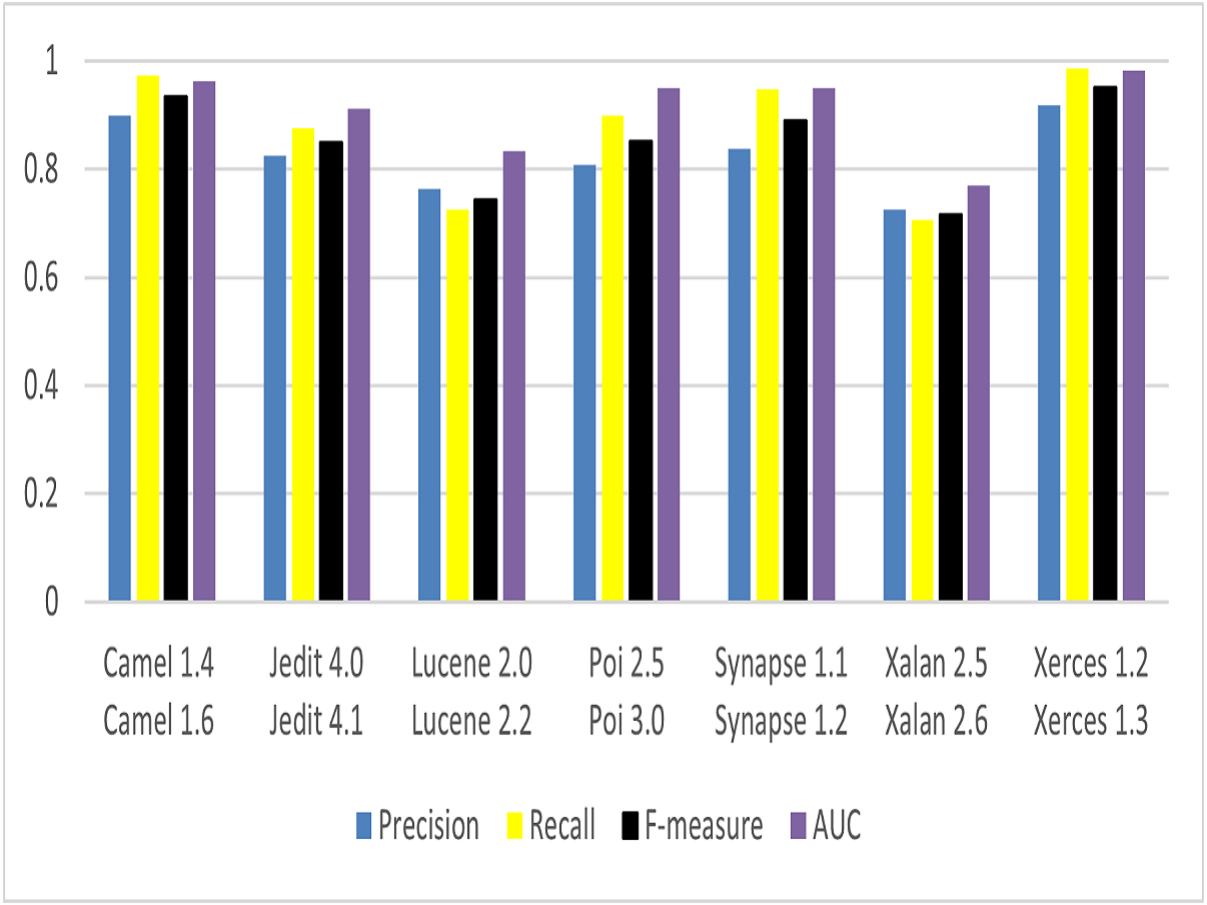
Performance of CBIL for WPDP.

**Figure 12:**
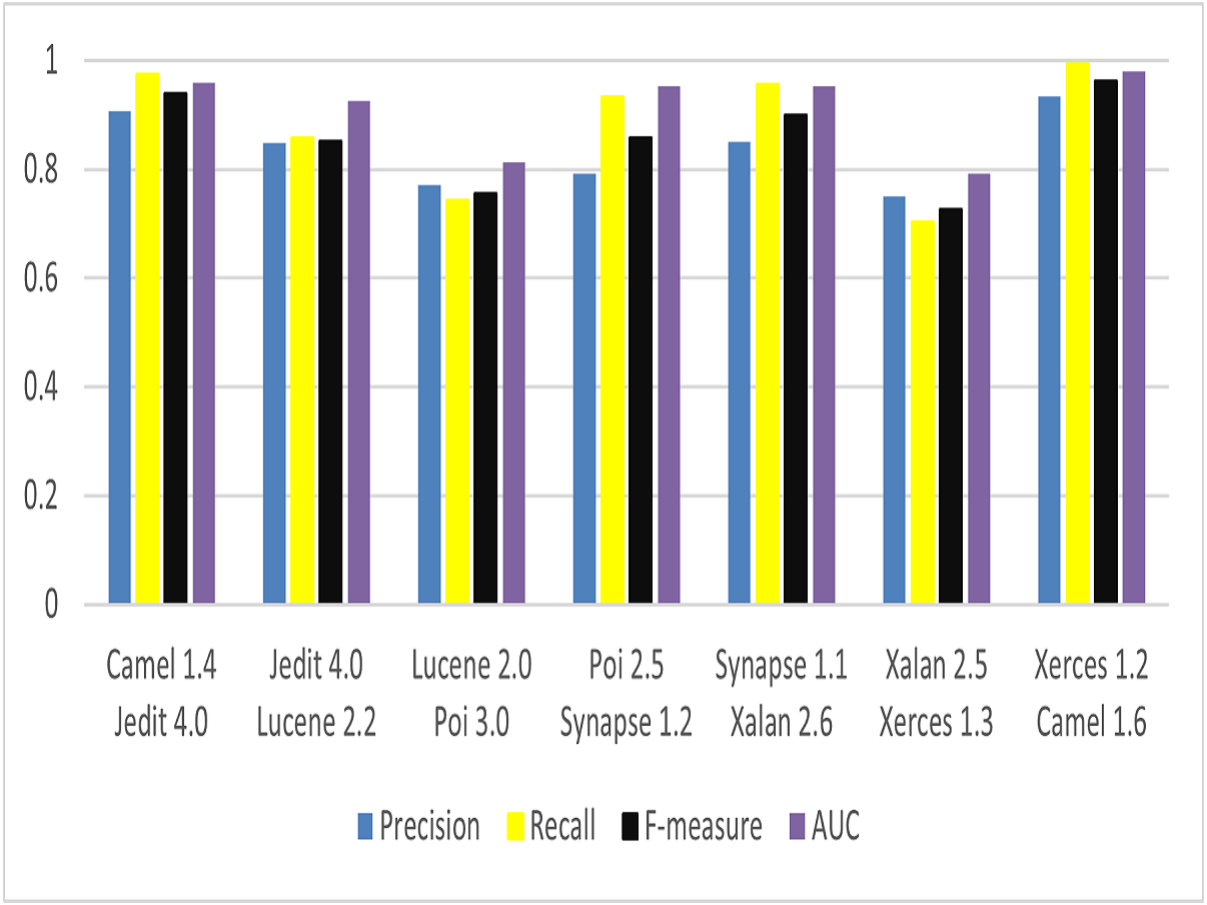
Performance of CBIL for CPDP.

To answer RQ4, to know the effectiveness of CBIL model on WPDP and CPDP, four evaluation metrics are chosen to evaluate the model. These metrics are Precision, Recall, *F*-measure, and AUC. The same parameter settings are used that discussed in ‘Environment and Experiments’. For WPDP, these metrics are applied on PROMISE dataset as shown before in Table 1. For each project, first release is used for training the model and second release for evaluating it. For example: Camel 1.4 is chosen to train the model, and Camel 1.6 for evaluating it. The result of the four-evaluation metrics is shown in Fig. 11. CBIL model achieves very good results for all metrics in WPDP. It achieves 0.825, 0.873, 0.848, and 0.908 for Precision, Recall, *F*-measure, and AUC respectively. For CPDP, the same metrics are chosen as in WPDP. A combination of projects is taken as samples for training set and test set as shown in Table 5. Source projects are used for training set and target projects are used for test set. The result is shown in Fig. 12. The proposed model again achieves good results for Precision, Recall, *F*-measure, and AUC as 0.813, 0.857, 0.833, and 0.899 respectively.

**Table 5:** Sample of PROMISE dataset for CPDP.

Source project	Target project
Camel 1.4	Jedit 4.0
Jedit 4.0	Lucene 2.2
Lucene 2.0	Poi 3.0
Poi 2.5	Synapse 1.2
Synapse 1.1	Xalan 2.6
Xalan 2.5	Xerces 1.3
Xerces 1.2	Camel 1.6

The results show that CBIL performs better than other baseline models for the following reasons:

1. The traditional machine learning models concentrate on specific traditional features and ignore the other features. The ignored features may be defect prone. Also, the traditional models fail to capture the semantics of source code. While CBIL automatically extracts the key semantics of source code.
2. Compared with the used deep learning models (DBN, CNN, RNN). CBIL combines CNN and Bi-LSTM. CNN can automatically learn the defect features. It extracts the key semantics based on number of filters and filter length. Also, Bi-LSTM can detect the information of long-term dependencies. It handles the sequential data in both forward and backward directions.
3. CBIL achieves good results for both Within-Project Defect Prediction (WPDP) and Cross-Project Defect Prediction (CPDP).

## Conclusion and Future Work

Recently, deep learning has mostly been used in the prediction of software defects. The hybrid model CBIL is presented in this research. It combines both of CNN and Bi-LSTM. Also, it helps in enhancing code review and software testing to predict the defective areas in source code. CBIL utilizes CNN to extract semantic features from AST tokens. Then, Bi-LSTM detects information of long-term dependencies to capture necessary features. CBIL model is evaluated on seven open-source Java projects from PROMISE dataset. The results display that CBIL improves the baseline models by 30% and improves CNN by 25% in average of *F*-measure for WPDP, as CNN is the best performance of all baseline models. In average of AUC, CBIL improves the baseline models by 21% and improves RNN by 18%, as RNN is the best performance of all baseline models. The proposed model CBIL achieves good results for both WPDP and CPDP. In the future, it is valuable to add quality metrics like defect density, types of defects, and defect severity. Also, CBIL can be applied on several open-source projects written in different programming languages. Moreover, more data preprocessing techniques could be added to enhance the quality of public datasets.

## Supplemental Information

10.7717/peerj-cs.739/supp-1 Supplemental Information 1 Python code of software defect prediction using the CBIL model and data

## Additional Information and Declarations

### Data Availability

The following information was supplied regarding data availability: The code and data are available in the Supplementary File.
